# Evaluating the Effect of Bile Acid Levels on Maternal and Perinatal Outcomes in Intrahepatic Cholestasis of Pregnancy: A Retrospective Study

**DOI:** 10.3390/diagnostics15172185

**Published:** 2025-08-28

**Authors:** Petra Gregorc, Ivan Verdenik, Polona Pečlin

**Affiliations:** 1 Division of Obstetrics and Gynaecology, Department of Perinatology, University Medical Centre Ljubljana, 1000 Ljubljana, Slovenia; petra.gregorc@zd-lj.si (P.G.); ivan.verdenik@guest.arnes.si (I.V.); 2 Department of Gynaecology and Obstetrics, Faculty of Medicine, University of Ljubljana, 1000 Ljubljana, Slovenia

**Keywords:** intrahepatic cholestasis of pregnancy, peak bile acid, adverse perinatal outcomes, stillbirth

## Abstract

**Background**: Intrahepatic cholestasis of pregnancy (ICP) is the most common reversible liver disorder linked to pregnancy, characterised by pruritus and elevated serum bile acids (BAs). Condition severity correlates with increased maternal and neonatal complications, and recent evidence highlights a significantly elevated risk of adverse perinatal outcomes, including stillbirth, when BA > 100 µmol/L. **Methods**: This retrospective study, conducted at a tertiary perinatology centre between 2019 and 2023, was performed in two phases. In the first phase, baseline group characteristics and pregnancy outcomes were compared between ICP and non-ICP (control) groups. In the second phase, outcomes were analysed across three ICP severity subgroups: mild (BA < 40 µmol/L), moderate (BA 40–99 µmol/L), and severe (BA ≥ 100 µmol/L). **Results**: A total of 210 patients diagnosed with ICP and 24,177 controls were included in the analysis. After multivariable regression, the results indicated that patients with severe ICP (BA ≥ 100 µmol/L) experienced significantly worse perinatal outcomes compared to those with mild or moderate disease: spontaneous preterm birth occurred in 26.7% of cases (*p* = 0.002), iatrogenic preterm birth in 36.7% (*p* < 0.001), meconium-stained amniotic fluid in 43.3% (*p* = 0.001), and neonatal intensive care unit (NICU) admission in 23.3% (*p* = 0.006). This subgroup also had the lowest mean birth weight (2830 g, *p* < 0.001). Notably, no stillbirths were recorded in any of the subgroups. Compared to controls, no major differences in maternal characteristics were noted, except in pregnancies conceived via in vitro fertilisation (IVF, *p* = 0.012) and those complicated by gestational diabetes (*p* = 0.040), both showing elevated risk for ICP development. **Conclusions**: This study confirms an association between ICP and increased perinatal complications, with severity of disease correlating with poorer outcomes. The findings highlight the need for standardised BA testing and improved strategies for perinatal management.

## 1. Introduction

Intrahepatic cholestasis of pregnancy (ICP) is the most common pregnancy-related reversible liver disorder. It is thought to result from a combination of hormonal, genetic, and environmental factors, and typically resolves spontaneously after delivery [1]. It commonly presents in the late second or third trimester, with pruritus and elevated serum bile acid (BA) concentrations [1,2]. Its prevalence varies globally from 0.5% to 25%, with higher rates reported in Scandinavian countries and a seasonal increase observed during winter months in regions such as Finland, Chile, and Portugal [3,4].

BAs are synthesised from cholesterol in the liver and play a critical role in the digestion and absorption of dietary fats. Due to their cytotoxicity at high levels, BA homeostasis is tightly regulated, primarily via the nuclear receptor farnesoid X receptor (FXR), whose activity is physiologically reduced during pregnancy. The foetal liver starts producing BAs at around 12 weeks gestation; however, their elimination relies on placental transfer to the maternal circulation. In ICP, the normal transplacental transport of BAs from foetus to mother is reversed, leading to their accumulation in the foetal circulation and contributing to placental dysfunction, increased uterine contractility, and foetal hypoxia. Elevated levels of total BAs are associated with oxidative stress and apoptosis, leading to damage of liver cells and other tissues [5,6].

Rising maternal BA levels are associated with increasingly adverse perinatal outcomes: levels exceeding 40 µmol/L are linked to elevated risks of preterm birth (PTB), meconium-stained amniotic fluid and foetal distress, while concentrations above 100 µmol/L have been associated with stillbirth. Although the exact mechanism is not known, elevated BA levels may trigger foetal arrhythmias or sudden vasoconstriction of placental vessels, contributing to unexpected intrauterine death. Neonatal respiratory distress syndrome has also been reported, likely due to the presence of BAs in the lungs [1,7,8].

Ursodeoxycholic acid (UDCA) remains the first-line treatment, with demonstrated benefits in alleviating maternal pruritus and potentially reducing the risk of PTB. However, current evidence regarding its effectiveness in preventing stillbirth remains inconclusive [5,7].

RCOG (Royal College of Obstetricians and Gynaecologists) and ACOG (American College of Obstetricians and Gynaecologists) guidelines recommend timing of delivery in ICP based on maternal BA levels, aiming to balance the risks of stillbirth and prematurity [2,9]. The 2025 FIGO (International Federation of Gynaecology & Obstetrics) guidelines incorporate the latest evidence and are designed for global implementation to enhance the management of pregnant women with pre-existing or newly diagnosed liver disease. They emphasise a multidisciplinary approach, appropriate diagnostic investigations, and timely treatment. They also include practical tables to support clinical decision-making [10]. Diagnosis of ICP is confirmed postpartum by the resolution of symptoms and normalisation of BA levels [9]. Accurate measurement of BAs, preferably fasting for diagnosis purposes and postprandial for assessing disease severity, is crucial for guiding management decisions [11,12].

This study examines the incidence, clinical characteristics, management strategies, and maternal and perinatal outcomes of pregnancies affected by ICP and compares them with outcomes of pregnancies without ICP. Additionally, it evaluates the association between maternal serum BA levels and outcomes in ICP pregnancies and identifies populations at increased risk of developing the condition.

## 2. Materials and Methods

This retrospective cohort study included all pregnant woman who gave birth at the Department of Perinatology, Division of Gynaecology, University Medical Centre Ljubljana, which is a tertiary care centre with over 5000 deliveries annually. This study was conducted between 1 January 2019 and 31 December 2023. Ethical approval was obtained from the Medical Ethics Committee of the University Medical Centre Ljubljana (approval no. KSEV-9-080425, 8 April 2025).

Data on pregnancy-related hepatopathies were obtained from a computerised medical record system and from the Slovenian National Perinatal Information System (NPIS) for a five-year period. NPIS is a population registry that registers all births at ≥22 weeks’ gestation or birth weight ≥500 g. Registration is mandatory by law, and more than 140 variables are entered immediately postpartum into a computerised database, which is regularly validated for accuracy. All patients diagnosed with ICP were identified using codes related to liver and biliary tract disorders in pregnancy (O26.6). Only those who underwent BA testing during pregnancy were included. Individual chart reviews were performed to confirm the diagnosis, defined by pruritus and a BA level >10 µmol/L. Individuals with other hepatobiliary conditions or metabolic syndromes were excluded. For patients with more than one ICP-affected pregnancy during the study period, each pregnancy was analysed as a separate case.

This study was conducted in two phases (Analysis 1 and Analysis 2).

**Figure 1:**
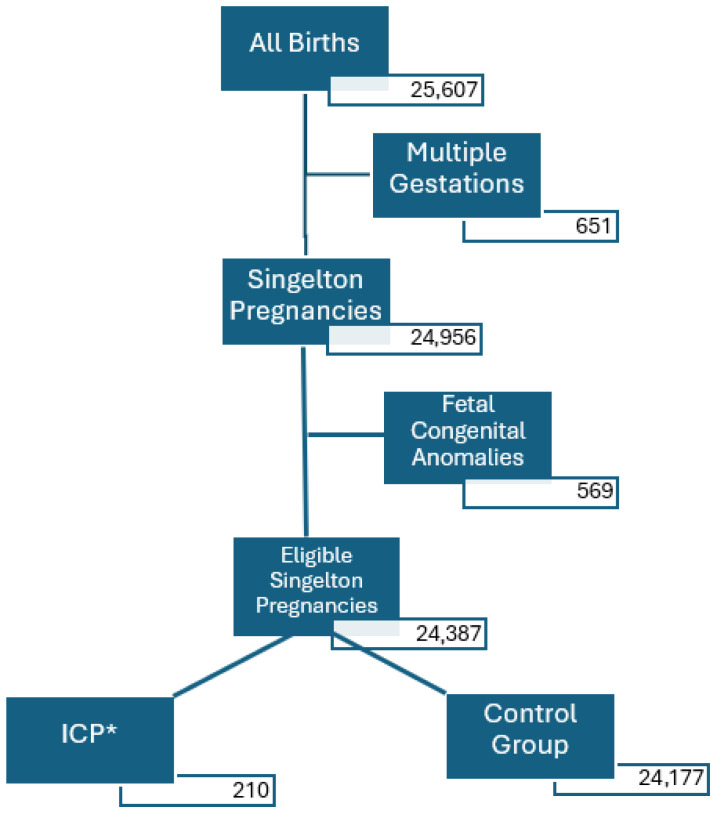
Flowchart of patient enrolment process for Analysis 1. * ICP—intrahepatic cholestasis of pregnancy.

In Analysis 1, patients were divided into two groups: the ICP group, which included pregnancies with an ICP diagnosis, and the control group, comprising pregnancies without an ICP diagnosis. Multiple gestations and patients with prenatally diagnosed foetal congenital anomalies were excluded (Figure 1).

**Figure 2:**
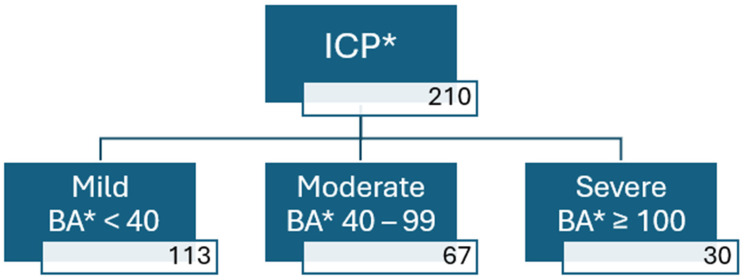
Flowchart of patient enrolment process for Analysis 2. * ICP—intrahepatic cholestasis of pregnancy, BA—peak serum bile acid concentration.

In Analysis 2, all patients diagnosed with ICP were further categorised into three subgroups according to their peak serum BA concentrations: mild (BA < 40 µmol/L), moderate (BA 40–99 µmol/L), and severe (BA ≥ 100 µmol/L) (Figure 2).

The variables assessed in both analyses are summarised in Table 1.

Ursodeoxycholic acid (e.g., Ursosan ^®^, PRO.MED.CS., Praha, Czech Republic) was administered at a daily dose of up to 15 mg/kg body weight, typically not exceeding six tablets per day, while cholestyramine (e.g., Questran ^®^, Cheplapharm Arzneimittel GmbH, Greifswald, Germany) was given at 4 g up to three times daily.

Statistical analyses were performed using IBM SPSS Statistics version 29, and a *p*-value of <0.05 was considered statistically significant. Continuous variables were presented as the mean ± standard deviation (SD), while categorical variables were presented as absolute numbers and percentages. While differences between groups were assessed using the Chi-square test, differences in means across multiple groups were evaluated using one-way ANOVA. To account for potential confounders and enhance the validity of the findings, multivariable logistic regression for binary outcomes and linear regression for continuous variables were applied, adjusting for maternal age, body mass index (BMI), smoking status, type 1 diabetes, gestational diabetes mellitus (GDM), IVF (in vitro fertilisation) conception, use of progesterone, season of birth, and foetal sex.

## 3. Results

Between 2019 and 2023, a total of 25,607 women gave birth at a tertiary perinatology centre. After exclusion of multiple gestations and foetal anomalies, 210 cases of ICP (0.82%) and 24,177 control pregnancies were included in the analysis (Figure 1).

In Analysis 1, comparison of baseline group characteristics between the ICP and control groups revealed no statistically significant differences in maternal age, parity, pregestational BMI, type 1 diabetes, foetal sex, use of progesterone, season of birth, or smoking history. However, statistically significant differences were observed in pregnancies conceived via IVF, and in the occurrence of GDM (Table 2).

After performing multivariable regression to adjust for confounders—including maternal age, BMI, smoking status, type 1-diabetes, GDM, IVF conception, use of progesterone, season of birth, and foetal sex—comparison of perinatal outcomes showed no statistically significant differences in the incidence of gestational hypertension, preeclampsia, emergency caesarean section, spontaneous PTB, intrapartum haemorrhage >500 mL, stillbirth, meconium-stained amniotic fluid, or Apgar score at 5 min. However, statistically significant differences were observed in gestational age at delivery, incidence of iatrogenic PTB, and elective caesarean section. Newborns of mothers with ICP had significantly lower birth weights compared to those in the control group. There was a trend toward higher NICU admission in the ICP group, although it did not reach statistical significance (Table 3).

In Analysis 2, among all women diagnosed with ICP (*n* = 210), 113 patients (54%) had a peak BA level of ≤40 µmol/L, 67 patients (32%) had peak BA levels between 40 and 99 µmol/L, and 30 patients (14%) had peak BA levels ≥100 µmol/L. Comparison of baseline characteristics between the groups revealed no statistically significant differences in any of the observed parameters (Table 4). Statistically significant differences between these groups were observed in gestational age at delivery, rates of spontaneous and iatrogenic PTB, presence of meconium-stained amniotic fluid, and admission to neonatal intensive care. Newborns born to mothers with higher peak BA levels also had progressively lower birth weights compared to those in the lower peak BA groups. No stillbirths occurred in any of the subgroups (Table 5).

Ursodeoxycholic acid was administered to 92 patients (80.7%) in the peak BA < 40 µmol/L group, 61 patients (91.0%) in the 40–99 µmol/L group, and all 30 patients (100%) in the ≥100 µmol/L group (*p* = 0.05). Cholestyramine was administered to 2 (1.8%), 7 (10.4%), and 13 patients (43.3%) in the peak BA < 40 µmol/L, 40–99 µmol/L, and ≥100 µmol/L groups (*p* < 0.001), respectively.

## 4. Discussion

This retrospective study aimed to evaluate pregnancy outcomes in women with ICP in relation to peak BA concentrations, while also providing a direct comparison to a large cohort of pregnancies without ICP. By stratifying ICP cases according to peak BA levels (<40, 40–99, and ≥100 µmol/L), our research uniquely integrates intergroup and intragroup analyses. To our knowledge, no previous study has combined these approaches, making this study well-positioned to offer a comprehensive perspective on ICP’s clinical implications.

Women with ICP delivered significantly earlier than those without the condition, with a mean gestational age of 37.21 weeks compared to 38.73 weeks in the control group. Among ICP cases, higher peak BA levels were associated with progressively earlier deliveries, with the BA ≥ 100 µmol/L subgroup delivering at a mean of 36.07 weeks. There were higher rates of both spontaneous (up to 26.7%) and iatrogenic PTBs (up to 36.7%) in this ICP subgroup, reinforcing the association between severe cholestasis and the risk of PTB. Recent advances in the etiological classification of PTB have reframed it as a multifactorial syndrome comprising distinct phenotypes defined by maternal, foetal, placental, and environmental conditions, rather than gestational age alone (Villar et al.) [13]. As noted in the taxonomy, the conventional distinction between spontaneous and medically indicated PTB presents a conceptual challenge, given the complex interplay of underlying pathophysiology and clinical decision-making. Within this framework, ICP, particularly in its severe form (BA levels ≥ 100 µmol/L), aligns with the Substantial Maternal Conditions phenotype, reflecting hepatic dysfunction with systemic consequences. ICP is associated with an increased likelihood of spontaneous PTB, likely driven by maternal disease processes, while obstetric management frequently involves planned early delivery to reduce the risk of stillbirth—accounting for the provider-initiated (iatrogenic) component observed in our cohort. The pattern of earlier delivery in severe ICP, shaped by both biological and clinical factors, may represent a subtype of PTB phenotype not yet fully characterised within current etiological models. Recognising ICP as an etiologically distinct phenotype may enhance risk stratification and inform more individualised approaches to perinatal surveillance and intervention.

**Figure 3:**
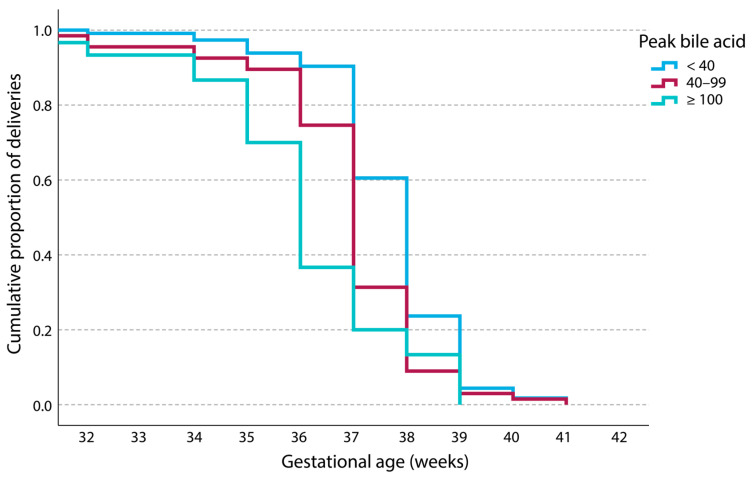
Cumulative Kaplan–Meier curves for gestational age at delivery. The graph depicts the cumulative percentage of three groups in relation to the peak bile acid values (in µmol/L).

**Figure 4:**
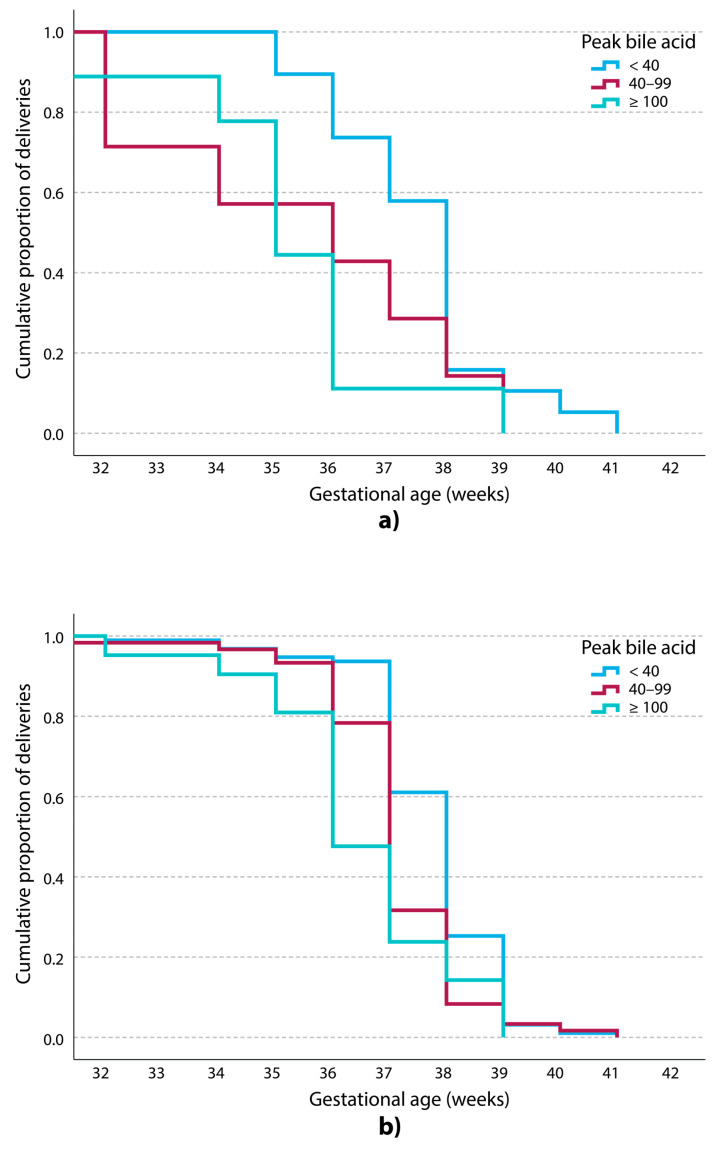
(**a**) Cumulative Kaplan–Meier curves for gestational age at delivery for spontaneous preterm births. The graph depicts the cumulative percentage of three groups in relation to the peak bile acid values (in µmol/L). (**b**) Cumulative Kaplan–Meier curves for gestational age at delivery for iatrogenic preterm births (induced labour and/or elective caesarean section). The graph depicts the cumulative percentage of three groups in relation to the peak bile acid values (in µmol/L).

Kaplan–Meier survival analysis (Figure 3 and Figure 4a,b) demonstrated significantly earlier gestational age at delivery with increasing BA levels.

Neonatal outcomes were notably worse among pregnancies with severe ICP (BA ≥ 100 µmol/L). This subgroup experienced significantly higher rates of NICU admissions, lower birth weights, and a higher incidence of meconium-stained amniotic fluid (43.3%) compared to those with lower BA levels. These findings suggest a clear relationship between elevated maternal BA concentrations and increased risk of adverse neonatal outcomes. These findings are consistent with the literature describing associations between ICP, PTB, and lower neonatal weight [9,14,15,16].

Although the RCOG and ACOG guidelines differ slightly in their recommendations regarding timing of delivery, both support delivery planning based on serum BA levels, particularly given the significantly increased risk of stillbirth at concentrations above 100 µmol/L [9]. In response to this risk, RCOG recommends induction between 35 and 36 weeks of gestation, while ACOG advises delivery at 36 + 0 weeks [2,9]. These recommendations account for the earlier timing of labour induction in women with severe ICP, thereby contributing to the observed reductions in gestational age and neonatal birth weight. However, the increased frequency of spontaneous PTB observed in this population suggests that underlying disease severity may also independently predispose to earlier delivery.

Several studies have linked ICP to comorbid conditions like GDM, pregnancy-induced hypertension, and preeclampsia [9,17,18,19]. Emerging evidence suggests that BAs may influence glucose and lipid homeostasis via FXRs, thereby contributing to insulin resistance [20,21]. Martineau et al.’s [22] study found higher GDM incidence in women predisposed to ICP, potentially due to shared or overlapping genetic and metabolic mechanisms. In our cohort, we observed similar associations between ICP and GDM; however, no statistically significant differences were found across BA subgroups.

Higher BMI has been investigated as a potential risk factor for ICP. While a Chinese study [23] reported no significant association, a Mexican study [24] found an increased prevalence of ICP in women with a pre-pregnancy BMI > 23 kg/m^2^. In our cohort, however, no significant differences in ICP incidence were observed across BMI groups.

Given the hormonal component of ICP pathophysiology, pregnancies conceived via IVF may carry an increased risk of developing the condition [25,26]. Bulkabas et al. [27] reported elevated BA levels in IVF conceived pregnancies without affecting ICP symptom onset, while Alemdaroglu et al. [28] found no differences in ICP outcomes based on mode of conception. In our cohort, a higher proportion of women with ICP underwent IVF (8.1% vs. 4.5%), a trend that was particularly evident in the BA ≥100 µmol/L group. In our setting, IVF pregnancies are monitored under the same standard surveillance protocols as spontaneous conceptions, and testing for ICP is performed under identical conditions. Therefore, the increased rate of ICP observed in IVF pregnancies is unlikely to be due to heightened diagnostic vigilance. However, we cannot exclude the possibility that other comorbidities associated with IVF pregnancies—excluding pre-existing liver disease, as women with such conditions were not included in the study—along with the influence of the hormonal environment typical of assisted reproduction, may contribute to this increased incidence.

Tsur et al. [29] showed that use of vaginal progesterone was linked to ICP. In our cohort, progesterone use was slightly more common in ICP pregnancies (9.5% vs. 8.4%), with a visible, though statistically insignificant, increase in the highest BA category.

ICP is not, in itself, an indication for caesarean delivery. Emergency and elective caesarean sections should be performed in accordance with standard obstetric indications and clinical guidelines [30]. In our cohort, a higher rate of elective caesarean deliveries was observed among women with ICP; however, peak BA levels did not appear to significantly influence the rate of caesarean sections or the frequency of vacuum-assisted deliveries. This is likely attributable to the increased incidence of both spontaneous and iatrogenic PTB, reaching up to 26.7% and 36.7%, respectively, in the subgroup with BA ≥ 100 µmol/L.

Stillbirth remains a major concern in ICP [9,16]. Although meta-analyses indicate a significantly increased risk, primarily when maternal BA levels reach or exceed 100 µmol/L [14], a residual risk may persist even at lower levels, including at levels <40 µmol/L, which is the commonly proposed threshold for elevated risk [15]. In our five-year cohort, no stillbirths were recorded in any subgroup, including those with peak BA ≥ 100 µmol/L. This outcome may be attributable, at least in part, to the high rate of PTB spontaneous or iatrogenic, observed in the ≥100 µmol/L group, where more than 60% of pregnancies ended before term. Kaplan–Meier survival analysis (Figure 3) highlights a leftward shift in the BA ≥ 100 µmol/L subgroup, indicating a clear tendency toward earlier delivery. Although foetal losses were not observed in the cohort overall, this shift suggests that proactive obstetric management, whether medically indicated or prompted by spontaneous PTB, may contribute to reducing the risk of stillbirth in cases of severe ICP (Figure 4a,b).

A meta-analysis of pregnancies complicated by ICP has demonstrated an increased risk of meconium-stained amniotic fluid, irrespective of BA levels [7,9]. The UKOSS study [9,18] further reported that meconium-stained amniotic fluid occurred even at earlier gestational ages, most frequently between 35 and 38 weeks, in women with BA > 40 µmol/L. When comparing pregnancies affected by ICP to those without the condition, our cohort demonstrated a trend toward an increased rate of meconium-stained amniotic fluid among ICP pregnancies (20.5% vs. 17.0%), although the overall difference did not reach statistical significance. Notably, this association became statistically significant in the subgroup with BA ≥ 100 µmol/L, where the rate reached 43.3%. This may serve as an early clinical indicator of foetal distress in severe ICP. The 43.3% occurrence of meconium-stained amniotic fluid in the BA ≥ 100 µmol/L group is strikingly high, especially considering that more than half of the newborns in this category were delivered preterm. In general, meconium-stained amniotic fluid is rare in preterm deliveries [31], as its occurrence typically increases with advancing gestational age due to greater foetal maturity and heightened susceptibility to intrauterine stress. The disproportionately elevated rate observed in severe ICP cases may indicate a distinct pathophysiological mechanism, potentially involving BA-mediated foetal distress that manifests even at earlier gestational stages. These findings support current clinical recommendations to induce labour between 35 and 36 weeks in this high-risk subgroup. However, our findings also indicate that, in many cases, labour would have commenced spontaneously around that time regardless.

A 5 min Apgar score <7 was more frequent in the subgroup with BA ≥ 100 µmol/L; however, after performing multivariable regression to adjust for confounders, this difference was no longer statistically significant. These findings align with results from a meta-analysis of over 5000 ICP cases [7,9], which also reported no significant association with low Apgar scores.

The UKOSS study [9,18] also reported that 45% of NICU admissions were due to prematurity and 30% due to respiratory issues. Similarly, in our study, NICU admission rates were elevated in the BA ≥ 100 µmol/L subgroup, where labour often began spontaneously or was induced prior to 37 weeks of gestation. The increased burden of prematurity-related complications in this subgroup likely contributed to the need for NICU care.

Seasonal variations in ICP incidence have been inconsistently reported. While Berg et al. [32] identified a peak incidence during the winter months in Scandinavia, Shahal et al. [33] observed a lower incidence of ICP in winter in Turkey. Several other studies have found no consistent seasonal pattern. Additionally, Bartolone et al. [34] investigated the influence of foetal sex on the course of ICP and associated outcomes, reporting no significant differences in incidence, severity, or pregnancy outcomes based on the sex of the neonate. Consistent with these findings, our analysis revealed no significant associations between neonatal outcomes and either season of delivery or foetal sex.

In our cohort, UDCA, the first-line pharmacological treatment for ICP [7,8], was frequently prescribed, primarily for the relief of maternal pruritus. While earlier studies have suggested that UDCA does not significantly improve perinatal outcomes or reduce stillbirth risk [7,35], more recent meta-analyses have reported a potential benefit. Specifically, UDCA use has been associated with a reduced risk of PTB, and when limited to randomised controlled trials, with a decreased combined risk of stillbirth and PTB. These findings support the clinical benefit of antenatal UDCA therapy [7]. In our cohort, 80% of women with BA levels <40 µmol/L received UDCA, while UDCA was administered to all women in the ≥100 µmol/L subgroup.

Unlike many studies that focus exclusively on pregnancies affected by ICP, our research offers a direct comparison with a large cohort of unaffected pregnancies (*N* = 24,177 vs. 210 ICP cases), while additionally stratifying ICP cases by peak maternal BA levels. As previously noted, to our knowledge, no prior research has simultaneously incorporated both a large-scale comparison with unaffected pregnancies and stratification of ICP cases by peak BA levels. This combined approach uniquely strengthens the clinical relevance and comprehensiveness of our findings on the spectrum of ICP severity.

BA concentrations fluctuate significantly throughout the day, particularly in response to food intake. In our study, BA levels were measured irrespective of fasting status or time of day, introducing potential intra-individual variability. This non-standardised sampling represents a limitation of our study, as it may have affected the comparability and interpretation of BA values across participants. Evidence from studies such as Huri et al. [11] suggests that fasting BA levels provide greater diagnostic specificity for ICP, whereas postprandial levels may better reflect disease severity. Accordingly, simultaneous assessment of both fasting and postprandial BA is recommended over random sampling. Future studies should examine pre- and postprandial levels and correlate these with pregnancy outcomes, potentially enabling the development of more precise diagnostic and management protocols.

The retrospective single centre design of this study represents a limitation, as findings may not be fully generalizable to broader populations or different clinical settings. Nevertheless, we consider the results relevant to other major obstetric centres, given that our institution is Slovenia’s largest tertiary care facility, accounting for nearly one-third of all national deliveries and managing a substantial number of referrals from across the country.

Importantly, this study’s strengths lie in its strict methodological design and rigorous inclusion criteria, focusing exclusively on patients with confirmed ICP and no concurrent liver disease. The use of multivariable regression to adjust for potential confounders further strengthens the validity of our findings, allowing for a more accurate assessment of associations between ICP and perinatal outcomes. Additionally, the relatively large sample size enhances the statistical reliability and robustness of the findings.

## 5. Conclusions

This study confirms an association between ICP and increased rates of maternal and neonatal complications. We observed a significantly higher incidence of spontaneous and iatrogenic PTBs, meconium-stained amniotic fluid, and NICU admissions, particularly among women with severe ICP (BA ≥ 100 µmol/L). Furthermore, women who conceived via IVF and those with GDM demonstrated an elevated risk of developing ICP, highlighting the importance of clinical vigilance and proactive management in these vulnerable populations.

While these findings provide important insights, further research is needed to define standardised criteria and optimal timing for BA testing, aiming to ensure consistent diagnostic practices and enhance outcomes in affected pregnancies.

## Figures and Tables

**Table 1:** Variables analysed in the study.

Baseline Characteristics of the Groups		
Maternal age	Primipara/multipara	GDM *
Smoking	Mode of conception: IVF *	Season of birth
Pregestational BMI *	DM type 1 *	Sex of newborn
**Maternal Treatment and Biochemical Markers**		
Use of progesterone	Use of cholestyramine	Use of ursodeoxycholic acid
Peak pregnancy bile acid levels		
**Maternal and Delivery Outcomes**		
Gestational age at delivery	Gestational hypertension	Preeclampsia
Caesarean section	Vacuum extraction delivery	Intrapartum haemorrhage >500 mL
Spontaneous preterm birth	Iatrogenic preterm birth **	
**Neonatal Outcome**		
Meconium-stained amniotic fluid	Apgar score <7 at 5 min ***	Umbilical artery pH
NCIU * admission	Birth weight	

* BMI—body mass index (kg/m^2^), IVF—in vitro fertilisation, DM type 1—diabetes mellitus type 1, GDM—gestational diabetes mellitus, NCIU—Neonatal intensive care unit. ** Iatrogenic preterm birth induced labour and/or elective caesarean section before 37 weeks of gestation. *** Apgar score at 5 min (good outcome: ≥7).

**Table 2:** Group characteristics in pregnancies with and without intrahepatic cholestasis (ICP).

	ICP Group (*N* = 210)	Control Group (*N* = 24,177)	*p*-Value (%)
Maternal age (years) mean (st. deviation)	31.62 (5.903)	31.10 (5.067)	0.133
Pregestational BMI, *n* (%) *			0.106
<18.5, *n* (%)	13 (6.2)	941 (3.9)	
18.5–25, *n* (%)	120 (57.1)	15,279 (63.3)	
25–30, *n* (%)	44 (21.0)	4982 (20.6)	
>30, *n* (%)	33 (15.7)	2951 (12.2)	
Smoking, *n* (%)	21 (10.0)	2451 (10.1)	0.947
DM1, *n* (%) *	1 (0.5)	141 (0.6)	0.839
IVF, *n* (%) *	17 (8.1)	1083 (4.5)	0.012
GDM, *n* (%) *	54 (25.7)	4836 (20.0)	0.040
Use of progesterone, *n* (%)	20 (9.5)	2024 (8.4)	0.549
Primipara, *n* (%)	97 (46.2)	11,326 (46.8)	0.850
Season of birth—winter, *n* (%) *	107 (51.0)	11,667 (48.3)	0.436
Foetal sex—male, *n* (%)	103 (49.0)	12,421 (51.4)	0.794

* BMI—body mass index (kg/m^2^), DM 1—diabetes mellitus type 1, GDM—gestational diabetes mellitus, IVF—in vitro fertilisation, Season of birth—winter—from October to March.

**Table 3:** Maternal and neonatal outcomes in pregnancies with and without intrahepatic cholestasis (ICP).

	ICP Group (*N* = 210)	Control Group (*N* = 24,177)	*p*-Value (%)	Adjusted *p*-Value (%) †
Gestational hypertension, *n* (%)	17 (8.1)	1594 (6.6)	0.383	0.641
Preeclampsia, *n* (%)	5 (2.4)	527 (2.2)	0.842	0.996
Gestational age at delivery (weeks) mean, (st. deviation)	37.21 (1.656)	38.73 (2.245)	<0.001	<0.001
Spontaneous preterm birth, *n* (%)	17 (8.1)	1209 (5.0)	0.041	0.055
Iatrogenic preterm birth, *n* (%) **	30 (14.3)	661 (2.7)	<0.001	<0.001
Caesarean section, *n* (%)	61 (29.0)	5038 (20.8)	0.004	0.012
Elective caesarean section, *n* (%)	32 (15.2)	2495 (10.3)	0.020	0.043
Emergency caesarean section, *n* (%)	22 (10.5)	1751 (7.2)	0.072	0.100
Vacuum extraction delivery, *n* (%)	8 (3.8)	586 (2.4)	0.195	0.193
Intrapartum haemorrhage > 500 mL, *n* (%)	15 (7.1)	1607 (6.6)	0.774	0.908
Meconium-stained amniotic fluid, *n* (%)	43 (20.5)	4115 (17.0)	0.185	0.183
Apgar 5 < 7, *n* (%) *	4 (1.9)	231 (1.0)	0.161	0.215
pH a. umbilicalis (*n*) mediana (st. deviation)	7.25 (0.077)	7.26 (0.082)	0.794	0.761
Stillbirth, *n* (%)	0 (0)	89 (0.4)	0.378	0.995
NCIU admission, *n* (%) *	20 (9.5)	1460 (6.0)	0.035	0.051
Birth weight (g) mean, (st. deviation)	3132.62 (507.642)	3340.57 (608.622)	<0.001	<0.001

† Adjusted for maternal age, BMI, smoking, DM type 1, GDM, IVF conception, use of progesterone, season of birth, and foetal sex. * Apgar 5 < 7—Apgar score after 5 min less than 7, NICU—neonatal intensive care unit. ** Iatrogenic preterm birth–induced labour and/or elective caesarean section before 37 weeks of gestation.

**Table 4:** Group characteristics in pregnancies with intrahepatic cholestasis stratified by peak bile acid levels.

Peak Bile Acid Level (µmol/L)	<40 (*N* = 113)	40–99 (*N* = 67)	≥100 (*N* = 30)	*p*-Value (%)
Maternal age (years) mean, (st. deviation)	31.75 (5.935)	31.34 (5.917)	31.77 (5.929)	0.896
Pregestational BMI, *n* (%) *				0.632
<18.5, *n* (%)	6 (5.3)	4 (6.0)	3 (10.0)	
18.5–25, *n* (%)	64 (56.6)	42 (62.7)	14 (46.7)	
25–30, *n* (%)	25 (22.1)	10 (14.9)	9 (30.0)	
>30, *n* (%)	18 (15.9)	11 (16.4)	4 (13.3)	
Smoking, *n* (%)	12 (10.6)	8 (11.9)	1 (3.3)	0.405
DM 1, *n* (%) *	0 (0)	1 (1.5)	0 (0)	0.342
GDM, *n* (%) *	29 (25.7)	19 (28.4)	6 (20.0)	0.684
IVF, *n* (%) *	5 (4.4)	7 (10.4)	5 (16.7)	0.064
Use of progesterone, *n* (%)	9 (8.0)	7 (10.4)	4 (13.3)	0.641
Primipara, *n* (%)	50 (44.2)	29 (43.3)	18 (60.0)	0.259
Season of birth—winter, *n* (%) *	63 (55.8)	29 (43.3)	15 (50.0)	0.269
Foetal sex—male, *n* (%)	57 (50.4)	30 (44.8)	16 (53.3)	0.671

* BMI—body mass index (kg/m^2^), DM 1—diabetes mellitus type 1, GDM—gestational diabetes mellitus, IVF—in vitro fertilisation, Season of birth—winter—from October to March.

**Table 5:** Maternal and neonatal outcomes in pregnancies with intrahepatic cholestasis stratified by peak bile acid levels.

Peak Bile Acid Level (µmol/L)	<40 (*N* = 113)	40–99 (*N* = 67)	≥100 (*N* = 30)	*p*-Value (%)	Adjusted *p*-Value (%) †
Gestational hypertension, *n* (%)	9 (8.0)	5 (7.5)	3 (10.0)	0.912	0.072
Preeclampsia, *n* (%)	3 (2.7)	2 (3.0)	0 (0)	0.646	0.131
Gestational age at delivery (weeks) mean, (st. deviation)	37.71 (1.321)	36.90 (1.698)	36.07 (1.982)	<0.001	<0.001
Spontaneous preterm birth, *n* (%)	5 (4.4)	4 (6.0)	8 (26.7)	<0.001	0.002
Iatrogenic preterm birth, *n* (%) **	6 (5.3)	13 (19.4)	11 (36.7)	<0.001	<0.001
Caesarean section, *n* (%)	37 (32.7)	16 (23.9)	8 (26.7)	0.428	0.146
Elective caesarean section, *n* (%)	22 (19.5)	7 (10.4)	3 (10.0)	0.183	0.217
Emergency caesarean section, *n* (%)	12 (10.6)	7 (10.4)	3 (10.0)	0.995	0.208
Vacuum extraction delivery, *n* (%)	4 (3.5)	3 (4.5)	1 (3.3)	0.941	0.701
Intrapartum haemorrhage > 500 mL, *n* (%)	6 (5.3)	6 (9.0)	3 (10.0)	0.529	0.795
Meconium-stained amniotic fluid, *n* (%)	16 (14.2)	14 (20.9)	13 (43.3)	0.002	0.001
Apgar 5 < 7, *n* (%) *	0 (0)	2 (3.0)	2 (6.7)	0.043	0.115
pH a. umbilicalis (*n*) mean (st. deviation)	7.26 (0.070)	7.25 (0.076)	7.22 (0.096)	0.016	0.215
Stillbirth, *n* (%)	0 (0)	0 (0)	0 (0)		
NCIU admission, *n* (%) *	6 (5.3)	7 (10.4)	7 (23.3)	0.011	0.006
Birth weight (g) mean (st. deviation)	3256.24 (505.857)	3059.33 (456.004)	2830.67 (478.993)	<0.001	<0.001

† Adjusted for maternal age, BMI, smoking, DM type 1, GDM, IVF conception, use of progesterone, season of birth, and foetal sex. * Apgar 5 < 7—Apgar score after 5 min less than 7, NICU—neonatal intensive care unit. ** Iatrogenic preterm birth–induced labour and/or elective caesarean section before 37 weeks of gestation.

## Data Availability

The authors serve as custodians of the anonymized data, which may be made available from the corresponding author upon reasonable request.
